# Comparison of toxicities between ultrahypofractionated radiotherapy versus brachytherapy with or without external beam radiotherapy for clinically localized prostate cancer

**DOI:** 10.1038/s41598-022-09120-0

**Published:** 2022-03-23

**Authors:** Hideya Yamazaki, Koji Masui, Gen Suzuki, Norihiro Aibe, Daisuke Shimizu, Takuya Kimoto, Kei Yamada, Akihisa Ueno, Toru Matsugasumi, Yasuhiro Yamada, Takumi Shiraishi, Atsuko Fujihara, Ken Yoshida, Satoaki Nakamura

**Affiliations:** 1 Department of Radiology, Kyoto Prefectural University of Medicine, 465 Kajiicho Kawaramachi Hirokoji, Kamigyo-ku, Kyoto, 602-8566 Japan; 2 Urology, Graduate School of Medical Science, Kyoto Prefectural University of Medicine, 465 Kajiicho Kawaramachi Hirokoji, Kamigyo-ku, Kyoto, 602-8566 Japan; 3 Department of Radiology, Kansai Medical University, Hirakata, 573-1010 Japan

**Keywords:** Oncology, Urology

## Abstract

To compare gastrointestinal (GI) and genitourinary (GU) toxicities in patients with localized prostate cancer treated with ultrahypofractionated radiotherapy (UHF) or brachytherapy [BT; low dose rate, LDR or high dose rate (HDR) with or without external beam radiotherapy (EBRT)]. We compared 253 UHF and 1664 BT ± EBRT groups. The main outcomes were the incidence and severity of acute and late GU and GI toxicities. The secondary endpoint was biochemical control rate. Cumulative late actuarial GU toxicity did not differ for grade ≥ 2 (8.6% at 5-years in UHF and 13.3% in BT ± EBRT, hazard ratio [HR], 0.7066; 95% CI, 0.4093–1.22, *p* = 0.2127). Actuarial grade ≥ 2 late GI toxicity was higher in UHF (5.8% at 5-years, HR: 3.619; 95% CI, 1.774–7.383, *p* < 0.001) than in BT ± EBRT (1.1%). In detailed subgroup analyses, the high-dose UHF group (H-UHF) using BED ≥ 226 Gy_1.5_, showed higher GI toxicity profiles than the other subgroups (HDR + EBRT, LDR + EBRT, and LDR monotherapy, and L-UHF BED < 226 Gy_1.5_) with equivalent GU toxicity to other modalities. With a median follow-up period of 32 months and 75 months, the actuarial biochemical control rates were equivalent between the UHF and BT ± EBRT groups. UHF showed equivalent efficacy, higher GI and equivalent GU accumulated toxicity to BT ± EBRT, and the toxicity of UHF was largely dependent on the UHF schedule.

## Introduction

Following the trend of shortening the treatment period in radiotherapy, stereotactic ablative body radiotherapy (SBRT), and high-precision external beam radiotherapy using strict image-guidance^1^ enabled us to perform ultrahypofractionated radiotherapy (UHF) using a single fraction dose of 5 Gy or more, which could reduce the burden on healthcare resources^1–5^. The biological features of prostate cancer with a low α/β ratio also encouraged the adoption of these hypo-to ultra-hypofractionation worldwide^1^. The recent HYPO-RT-PC randomized control trial provided evidence that UHF is non-inferior to standard conventional fractionation of 78 Gy in 2 Gy fractions^2^. Long-term^3^ and large cohort outcomes including meta-analysis from Western countries confirmed the efficacy of UHF^4,5^. However, selection of the best treatment option for patients with localized prostate cancer remain difficult due to the many curative treatment options, such as surgery, external beam radiotherapy, and brachytherapy (BT)^6^. BT is an established treatment for localized prostate cancer with excellent dose distribution, including low-dose rate (LDR) BT and high dose rate (HDR) BT^7^. BT can be administered as monotherapy (usually for low or lower titer intermediate-risk prostate cancer) or as a boost (for higher titers intermediate or high-risk prostate cancer). There are concerns that for intermediate-and high-risk disease, BT alone may not adequately treat the peri-prostatic tissues; therefore, BT has been used as a boost in combination with external beam radiotherapy (EBRT) for high-intermediate or high-risk prostate cancer in general^7^. Although several randomized controlled trials indicated the superiority of BT boost over external beam radiotherapy alone^8–10^ not only in LDR^8^ but also in HDR^9,10^, there is a lack of conclusive data comparing BT ± EBRT and UHF^11–15^. Therefore, to compare the results of UHF to BT ± EBRT, we used open data constructed by multi-institution data accumulation in Japan^16^. In addition, as previous studies cited that a BED over 226 Gy_1.5_ might be a threshold to cause higher rates of grade > 2 toxicities^16,17^ in UHF, we divided the UHF group into L-UHF (BED < 226 Gy_1.5_) and H-UHF (BED ≥ 226 Gy_1.5_) groups using this threshold. Then, we performed a subgroup analysis (LDR monotherapy, LDR + EBRT, HDR + EBRT, L-UHF, H-UHF) compared to BT ± EBRT versus UHF. Thus, the aim of the present study was to compare the toxicity and preliminary PSA control of UHF and BT ± EBRT.

## Methods

### Patients

We retrospectively examined 253 patients treated with UHF (open data for public use)^18^ and 1664 patients treated with BT ± EBRT (1161 HDR + EBRT from open data and 477 LDR ± EBRT treated at Kyoto Prefectural University of Medicine) (Table 1). The patient eligibility criteria included treatment with UHF or BT ± EBRT, stage T1–T3, and N0M0 with histology-proven adenocarcinoma; the availability and accessibility of pretreatment data (initial prostate-specific antigen = iPSA) level, Gleason score sum (GS), and T classification to determine the stage according to the NCCN 2015 risk classification as follows: low (T1–T2a, GS 2–6, and iPSA < 10 ng/mL), intermediate (T2b–T2c, GS 7, or PSA 10–20 ng/mL), and high (T3, GS 8–10, or PSA > 20 ng/mL)^19^. We excluded (1) node-positive cases, (2) metastasis cases, and (3) follow-up period of less than 20 months.

**Table 1:** Patients characteristics between ultrahypo fractionated radiotherapy and brachytherapy with or without external beam radiotherapy.

Variables	Group	UHF (n = 253)	Subgroup of UHF		BT ± EBRT (n = 1664)	Subgroup of BT ± EBRT			*p-*value*
			L-UHF (n = 162)	H-UHF (n = 91)		HDR + EBRT (n = 1187)	LDR (n = 411)	LDR + EBRT (n = 66)	
Age		72.00 [54.00, 86.00]	72.00 [54.00, 86.00]	73.00 [54.00, 86.00]	69.00 [42.00, 86.00]	70.00 [42.00, 86.00]	69.00 [45.00, 83.00]	68.00 [52.00, 79.00]	** < 0.001**
iPSA (mg/ml)		8.12 [1.70, 188.00]	7.81 [1.70, 87.60]	9.60 [3.90, 188.00]	10.70 [1.40, 3208.00]	14.72 [2.68, 3208.00]	6.80 [1.40, 26.00]	7.94 [3.20, 46.00]	** < 0.001**
T (%)	T1	97 (38.3)	69	28	472 (28.4)	240 (20.2)	220 (53.5)	12 (18.2)	** < 0.001**
	T2	131 (51.8)	86	45	641 (38.5)	407 (34.3)	191 (46.5)	43 (65.2)	
	T3	25 ( 9.9)	7	18	551 (33.1)	540 (45.5)	0 ( 0.0)	11 (16.7)	
GS (%)	≤ 6	56 (22.1)	44 (27.2)	12 (13.2)	373 (22.4)	101 ( 8.5)	263 (64.0)	9 (13.6)	**0.003**
	7	140 (55.3)	91 (56.2)	49 (53.8)	741 (44.5)	560 (47.2)	148 (36.0)	33 (50.0)	
	8 ≤	57 (22.5)	27 (16.7)	30 (33.0)	550 (33.1)	526 (44.3)	0 ( 0.0)	24 (36.4)	
NCCN (%)	High	67 (26.5)	30 (19.6)	37 (37.0)	939 (56.5)	901 (76.1)	2 ( 0.5)	36 (54.5)	** < 0.001**
	Intermediate	153 (60.5)	104 (64.2)	49 (53.8)	519 (31.2)	272 (23.0)	217 (52.8)	30 (45.5)	
	Low	33 (13.0)	28 (17.3)	5 ( 5.5)	203 (12.2)	11 ( 0.9)	192 (46.7)	0 ( 0.0)	
Follow-up periods	(Months)	32.00 [22.00, 97.00]	30.70 [22.00, 97.00]	36.00 [24.00, 77.00]	75.00 [22.00, 177.00]	69.00 [22.00, 177.00]	91.00 [29.00, 169.00]	78.00 [30.00, 148.00]	** < 0.001**
ADT (%)	Yes	149 (58.9)	83 (51.2)	66 (72.5)	1524 (91.6)	1134 (95.5)	330 (80.3)	60 (90.9)	** < 0.001**
	No	104 (41.1)	79 (48.8)	25 (27.5)	140 ( 8.4)	53 ( 4.5)	81 (19.7)	6 ( 9.1)	
Total ADT duration	(Months)	12.00 [2.00, 51.00]	2.50 [2.00, 48.00]	8.00 [2.00, 51.00]	32.00 [1.00, 112.00]	43.00 [1.00, 112.00]	6.00 [1.00, 24.00]	4.00 [1.00, 24.00]	**0.002**
Neo ADT (%)	Yes	143 (56.5)	77 (47.5)	66 (72.5)	1516 (91.1)	1127 (94.9)	329 (80.0)	60 (90.9)	** < 0.001**
	No	110 (43.5)	85 (52.5)	25 (27.5)	148 ( 8.9) |	60 ( 5.1)	82 (20.0)	6 ( 9.1)	
Neo. duration	(Months)	6.00 [1.00, 48.00]	6.00 [1.00, 48.00]	6.00 [3.00, 24.00]	8.00 [1.00, 92.00]	11.00 [1.00, 92.00]	6.00 [1.00, 24.00]	4.00 [1.00, 13.00]	** < 0.001**
Adjuvant ADT (%)	Yes	81 (32.0)	41 (25.3)	40 (44.0)	1089 (65.4)	1134 (95.5)	330 (80.3)	60 (90.9)	** < 0.001**
	No	172 (68.0)	121 (74.7)	51 (56.0)	575 (34.6)	53 ( 4.5)	81 (19.7)	6 ( 9.1)	
Adjuvant duration	(months)	18.00 [1.00, 39.00]	1.00 [3.00, 30.00]	24.00 [1.00, 39.00]	36.00 [1.00, 93.00]	36.00 [1.00, 93.00]	3.00 [1.00, 9.00]	3.00 [1.00, 19.00]	** < 0.001**

Bold values indicate statistically significance.

*p-value was calculated between UHF and BT ± EBRT.

*BT* brachytherapy, *EBRT* external beam radiotherapy, *UHF* ultrahypofractionated radiotherapy, *L-UHF* low dose UHF EQD2 < 100 Gy1.5 (α/β = 1.5), *H-UHF* high dose UHF EQD2 ≥ 100 Gy1.5 (α/β = 1.5).

The Common Terminology Criteria for Adverse Events version 4.0, was used for the toxicity analysis. Toxic effects occurring within 90 days after radiotherapy completion were considered acute, and toxic effects occurring after the 90-day period were considered late. Biochemical failure was defined as the time from the initiation of radiotherapy to the date of last follow-up and/or biochemical failure, whichever came first, according to the Phoenix definition (nadir, + 2 ng/ml)^19^.

All patients at Kyoto Prefectural University of Medicine provided written informed consent, and patients undergoing UFH (open data) and a part of those undergoing BT ± EBRT (open data) provided informed consent during the process of building public data. This study was conducted in accordance with the Declaration of Helsinki and with the permission of the Institutional Review Board (Kyoto Prefectural University of Medicine: ERB-C-1403).

### Treatment planning

#### LDR-BT with or without EBRT

**Figure 1:**
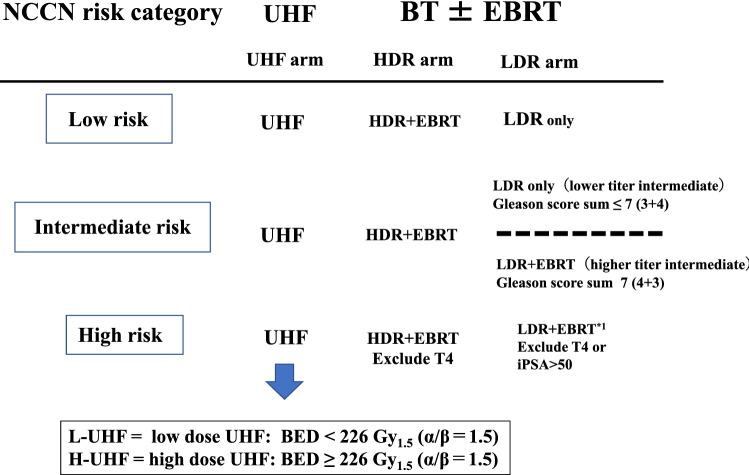
Scheme of treatments according to National Comprehensive Cancer Network (NCCN) risk classification. Abbreviations; *BT* brachytherapy, *HDR* high-dose-rate, *LDR* low-dose-rate, *EBRT* external beam radiotherapy, *UHF* ultrahypofractionated radiotherapy, *L-UHF* low dose UHF EQD2 < 100 Gy1.5 (α/β = 1.5), *H-UHF* high dose UHF EQD2 ≥ 100 Gy1.5 (α/β = 1.5).

The implant technique has been described in detail previously^20^. All patients underwent transrectal ultrasound preplanning 3–4 weeks before implantation to determine the number of seeds. Permanent intraoperative Iodine-125 implantation using a modified peripheral loading method. We used combination therapy (LDR + EBRT) for T3 or Gleason score sum ≤ 8, or Gleason score sum 7 (4 + 3) cases (not for Gleason score sum 7 (3 + 4) cases) (Fig. 1). The prescription dose for the clinical target volume (prostate) was 145 Gy (LDR alone) or 110 Gy [LDR with 40 Gy/ 20 fractions EBRT by three-dimensional conformal radiotherapy (3D-CRT)].

#### HDR-BT with EBRT

The multi-institution data were obtained from an open data source^18^, and the detailed method of applicator implantation has been described elsewhere^21^. All patients were treated with a combination of HDR and EBRT at various fractionations (Table 2). The median dose of HDR used was 31.5 Gy (10.5–31.5 Gy) and that of EBRT was 30 Gy (30–51 Gy). The median fraction size of HDR was 6.3 Gy (5–11 Gy) and that of EBRT was 3 Gy (2–3 Gy). Patients who were administered EBRT comprised 1166 (98.2%) on 3D-CRT and 21 (1.8%) on IMRT.

**Table 2:** Detailed treatment schedule.

Group	Subgroup	Prescribed dose/fraction No	PTNO	BED (total) (α/β = 1.5)(Gy)
Ultrahypofractionated radiotherapy (UHF)	Low dose UHF (L-UHF)	35 Gy/5fr (CyberKnife)	63	198
		32 Gy/4fr (CyberKnife)	9	202
		36.25 Gy/5fr (CyberKnife)	81	214
		34 Gy/4fr (Tomotherapy)	9	226
	High dose UHF (H-UHF)	36 Gy/4fr (Tomotherapy)	91	252
Brachytherapy (BT) ± external beam radiotherapy (EBRT)	High-dose rate (HDR)	HDR 10.5 Gy/2fr + EBRT 51 Gy/17 fr	1	237
		HDR 11 Gy/1fr + EBRT 51 Gy/17fr	129	245
		HDR 11 Gy/1fr + EBRT 45 Gy/15 fr	22	227
		HDR 18 Gy/2 fr + EBRT 39 Gy/13 fr	146	243
		HDR 18 Gy/2fr + EBRT 48 Gy/16fr	2	270
		HDR 18 Gy/2 fr + EBRT51 Gy/17fr	136	279
		HDR 20 Gy/2fr + EBRT 30 Gy/15 fr	1	223
		HDR 20 Gy/ 2fr + EBRT 46 Gy/23fr	18	260
		HDR 21 Gy/2 fr + EBRT 51 Gy/ 17 fr	1	321
		HDR 21 Gy/3 fr + EBRT 51 Gy/17 fr	18	272
		HDR 21 Gy/2fr + EBRT 42 Gy/14fr	2	294
		HDR 21 Gy/2 fr + EBRT 45 Gy/15fr	42	303
		HDR 25 Gy/5fr + EBRT 51 Gy/17 fr	9	261
		HDR 31.5 Gy/5fr + EBRT 30 Gy/10fr	660	253
	Low-dose –rate (LDR)	LDR 145 Gy	411	154
		LDR 110 Gy + EBRT 40 Gy /20fr	66	209

BED = nd(1 + d/[α/β]): *n* Number of treatment fractions, *d* Dose per fraction in Gy, α/β = 1.5, *BT* brachytherapy, *EBRT* external beam radiotherapy, *UHF* ultrahypofractionated radiotherapy, *HDR* high-dose-rate, *LDR* low-dose-rate, *L-UHF* low dose UHF BED < 226 Gy_1.5_ (α/β = 1.5), *H-UHF* high dose UHF BED ≥ 226 Gy_1.5_ (α/β = 1.5).

#### UHF

The detailed method of this study has been described elsewhere^16,22^. The median dose of UHF used was 36 Gy (32–36.25 Gy) and the median fraction size of UHF was 7.25 Gy (7–9 Gy) (Table 2).

### Statistical analysis

The R stat package^23^ was used for the statistical analyses. We analyzed percentages using chi-square tests. To compare medians or means, we used Mann–Whitney U-tests for skewed data and Student’s t-tests for normally distributed data^23^. To analyze the biochemical control rate, overall survival, and toxicity, we used Kaplan–Meier method and log-rank tests including Bonferroni test in pos t-hoc *p*-value adjustment was used^23^. Univariate and multivariate analyses were made with Cox’s proportional hazards model^23^. All analysis used statistical significance level set at *p* < 0.05.

We divided the UHF group into two subgroups according to previous studies^16,17^: high (H-UHF) and low dose UHF (L-UHF) groups, using a cut-off value of BED of 226 Gy_1.5_; BED = n × d × (1 + d/[α/β]) where d = dose per fraction in Gy, n = number of treatment fractions, α/β = 1.5.

Since the included patients were not randomized, unbalanced patients baseline characteristics could influence on the selection bias and, hence, influence the decision to undergo BT ± EBRT or BT. The propensity score was defined as the probability of allocation to the BT ± EBRT or UHF group, given the patient characteristics^23^. We used logistic regression model in the calculation of the propensity scores using the baseline covariates shown in Table 2.

We used a propensity score-matched pair analysis to reduce the bias for choice of treatment; the UHF or BT ± EBRT groups (total population and HDR + EBRT group). Five factors prescribed before were selected as the variables that would be significantly related to the decision to choose UHF or BT ± EBRT, and a 1:1 matched cohort was made. Same procedure was applied in comparison between UHF and HDR + EBTT.

## Results

### Patient and tumor characteristics

The baseline patient characteristics of the UHF and BT ± EBRT groups are shown in Table 1. The 1921 patients with stage T1–T3 N0M0 prostate cancers were treated using UHF or BT ± EBRT. The median patient age was 70 years (range, 42–86 years). The median follow-up duration for the entire cohort was 70 months (range, 22–177 months). BT ± EBRT was used to treat patients with advanced disease and hormonal therapy history with longer follow-up periods than those in the UHF group.

### Toxicity Comparison between UHF and BT ± EBRT

Table 3 shows the incidence of maximal grade of early and late gastrointestinal (GI) and genitourinary (GU) toxicities. UHF showed higher maximal grade GI and lower maximal grade GU toxicity than the BT ± EBRT group.

**Table 3:** Comparison of toxicity grade between UHF and BT.

Grade	UHF		Subgroup of UHF				BT ± EBRT		Subgroup of BT ± EBRT						*p-*value*
			L-UHF		H-UHF				HDR + EBRT		LDR		LDR + EBRT		
	( n = 253)	(%)	(n = 162)	(%)	(n = 91)	(%)	(n = 1664)	(%)	(n = 1187)	(%)	(n = 411)	(%)	(n = 66)	(%)	
**(a) Acute toxicity**															
Gastrointestinal No. (%)															
0	185	(73%)	146	(90%)	39	(43%)	1477	(89%)	1060	(89%)	369	(90%)	48	(73%)	** < 0.001**
1	55	(22%)	14	(9%)	41	(45%)	181	(11%)	123	(10%)	42	(10%)	16	(24%)	
2	13	(5%)	2	(1%)	11	(12%)	5	(0.3%)	3	(0.3%)	0	(0%)	2	(3%)	
3	0	(0%)	0	(0%)	0	(0%)	1	(0.1%)	1	(0.1%)	0	(0%)	0	(0%)	
Genitourinary No. (%)															
0	126	(50%)	99	(61%)	27	(30%)	470	(28%)	347	(29%)	33	(8%)	4	(6%)	
1	93	(37%)	43	(27%)	50	(55%)	865	(52%)	632	(53%)	204	(50%)	30	(45%)	** < 0.001**
2	34	(13%)	20	(12%)	14	(15%)	324	(19%)	119	(10%)	173	(42%)	32	(48%)	
3	0	(0%)	0	(0%)	0	(0%)	5	(0.3%)	4	(0.3%)	1	(0.1%)	0	(0%)	
**(b) Late toxicity**															
Gastrointestinal No. (%)															
0	206	(81%)	151	(93%)	55	(60%)	1419	(85%)	993	(83%)	378	(92%)	48	(73%)	**0.0181**
1	36	(14%)	9	(6%)	27	(30%)	207	(12%)	163	(14%)	29	(7%)	15	(23%)	
2	9	(4%)	2	(1%)	7	(8%)	37	(2%)	30	(3%)	4	(1%)	3	(5%)	
3	2	(1%)	0	(0%)	2	(2%)	1	(0.1%)	1	(0.1%)	0	(0%)	0	(0%)	
Genitourinary No. (%)															
0	170	(67%)	131	(81%)	39	(43%)	728	(44%)	534	(45%)	169	(41%)	25	(38%)	** < 0.001**
1	69	(27%)	27	(17%)	42	(46%)	671	(40%)	473	(40%)	168	(41%)	30	(45%)	
2	13	(5%)	4	(2%)	9	(10%)	158	(9%)	105	(9%)	69	(17%)	11	(17%)	
3	1	(0.1%)	0	(0%)	1	(1%)	80	(5%)	75	(6%)	5	(1%)	0	(0%)	

*BT* brachytherapy, *EBRT* external beam radiotherapy, *UHF* ultrahypofractionated radiotherapy, *HDR* high-dose-rate, *LDR* low-dose-rate, *L-UHF* low dose UHF EQD2 < 100 Gy1.5 (α/β = 1.5), *H-UHF* high dose UHF EQD2 ≥ 100 Gy1.5 (α/β = 1.5), *GU* genitourinary, *GI* gastrointestinal.

*p-value was calculated between UHF and BT ± EBRT.

**Figure 2:**
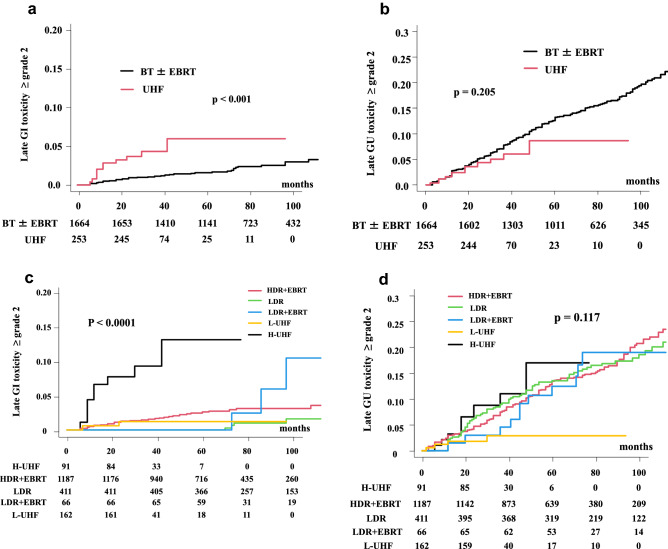
Comparison of accumulated incidence toxicity grade ≥ 2. (**a**) Accumulated incidence of grade ≥ 2 Gastrointestinal (GI) toxicity between BT ± EBRT and UHF. (**b**) Accumulated incidence of grade ≥ 2 Genitourinary (GU) toxicity between BT ± EBRT and UHF. (**c**) Accumulated incidence of grade ≥ 2 GI toxicity among subgroups. (HDR + EBRT vs. LDR + EBRT vs. DR monotherapy vs. L-UHF vs. H-UHF). (**d**) Accumulated incidence of grade ≥ 2 GU toxicity among subgroups. (HDR + EBRT vs. LDR + EBRT vs . LDR monotherapy vs. L-UHF vs. H-UHF).

The 3- (and 5-year) cumulative incidence of grade ≥ 2 GI toxicities was 4.2% (5.8%) in the UHF group and 1.1% (1.8%) in the BT ± EBRT group (*p* < 0.0001; Fig. 2a), with a hazard ratio of 3.661 (95% CI: 1.799–7.454, *p* < 0.0001).

The 3-year and 5-year cumulative incidence rates of grade ≥ 2 GU toxicities were 6.0% (8.6%) in the UHF group and 8.8% (13.3%) in the BT ± EBRT group (*p* = 0.205; Fig. 2b), with a hazard ratio of 0.7044 (95% CI: 0.408–1.216, *p* = 0.2085).

As shown in Table 4, the predictors of late GI toxicity grade ≥ 2 on the multivariate Cox regression analysis included modality (UHF worse than BT ± EBRT, hazard ratio 2.37, 95% CI = 1.04–5.39, *p* = 0.04), and acute GI toxicity grade ≥ 2 (hazard ratio 6.76, 95% CI = 1.94–23.59, *p* = 0.0027). For GU toxicity, only acute GU toxicity grade ≥ 2 (hazard ratio 2.19, 95% CI = 1.69–2.84, *p* < 0.0001) was identified as a statistically significant predictor of late GU toxicity grade ≥ 2.

**Table 4:** Multi-variate analysis for late GU and GI toxicity grade 2 ≤ using Cox proportional hazards model.

Variable	Strata	GI			GU		
		HR	95% CI	*p-*value	HR	95% CI	*p-*value
Age, years	≤ 70	1	(Referent)	–	1	(Referent)	–
	71 ≤	1.31	0.74–2.33	0.35	1.14	0.74–1.75	0.56
T classification	≤ 2	1	(Referent)	–	1	(Referent)	–
	3 ≤	0.82	0.38–1.78	0.62	0.8	0.59–1.08	0.15
Gleason score	≤ 7	1	(Referent)	–	1	(Referent)	–
	8 ≤	0.7	0.33–1.47	0.34	1.12	0.85–1.48	0.43
Pretreatment PSA (ng/mL)	≤ 10	1	(Referent)	–	1	(Referent)	–
	10 <	1.23	0.67–2.24	0.51	1.18	0.91–1.52	0.2
Hormonal therapy	No	1	(Referent)	–	1	(Referent)	–
	Yes	0.64	0.29–1.42	0.27	1.14	0.74–1.75	0.56
Acute toxicty grade 2 ≤	No	1	(Referent)	–	1	(Referent)	–
	Yes	6.76	1.94–23.59	**0.0027**	2.19	1.69–2.84	** < 0.0001**
Treatment modalities	BT ± EBRT	1	(Referent)	–	1	(Referent)	–
	UHF	2.37	1.04–5.39	**0.04**	0.76	0.43–1.35	0.35

Bold values indicate statistically significance.

Abbreviations; *CI* confidence interval, *HR* hazard ratio, *NA* not available, *DeRT* dose escalated radiotherapy, *UHF* ultrahypofractionated radiotherapy, *BT* brachytherapy, *EBRT* external beam radiotherapy, *UHF* ultrahypofractionated radiotherapy.

### Subgroup analysis for toxicity

In the detailed subgroup analysis, BT ± EBRT was divided into HDR + EBRT, LDR only, and LDR + EBRT, while UHF was divided into U-UHF and L-UHF (Table 2).

The 3-year and 5-year cumulative incidences of grade ≥ 2 late GI toxicities were 1.5% (2.6%), 0.3% (0.3%), 2.4% (2.4%), 1.2% (1.2%), and 9.3% (13%) in the HDR + EBRT, LDR only, LDR + EBRT, L-UHF, and H-UHF groups, respectively (*p* < 0.0001, Fig. 2c). H-UHF showed a higher cumulative incidence of GI toxicity than the other modalities (Table 5).

**Table 5:** Toxicity comparison among subgroup Patients characteristics between and BT with or without external beam radiotherapy.

Group	UHF		BT ± EBRT		
Subgroup (Accumulated incidence at 5-years) (PT NO)	L-UHF	H-UHF	HDR + EBRT	LDR	LDR + EBRT
	(1.2%)	(9.3%)	(1.5%)	(0.3%)	(2.4%)
	(n = 162)	(n = 91)	(n = 1187)	(n = 411)	(n = 66)
**(a) Gastrointestinal oxcicity**					
L-UHF	–	**0.0281**	1	0.0725	1
H-UHF	–	-	** < 0.001**	** < 0.001**	** < 0.001**
HDR + EBRT	–	–	–	0.0771	1
LDR	–	–	–	–	0.0891
LDR + EBRT	–	–	–	–	–

Group	UHF		BT ± EBRT		
Subgroup (Accumulated incidence at 5-years) (PT NO)	L-UHF	H-UHF	HDR + EBRT	LDR	LDR + EBRT
	(3.0%)	(11.1%)	(13.5%)	(13.5%)	(13.4%)
	(n = 162)	(n = 91)	(n = 1187)	(n = 411)	(n = 66)
**(b) Genitourinary toxicity**					
L-UHF	–	0.069	0.171	0.069	0.812
H-UHF	–	–	1	1	1
HDR + EBRT	–	–	–	1	1
LDR	–	–	–	–	1
LDR + EBRT	–	–	–	–	–

Bold values indicate statistically significance.

*BT* brachytherapy, *HDR* high-dose-rate, *LDR* low-dose-rate, *EBRT* external beam radiotherapy, *UHF* ultrahypofractionated radiotherapy, *L-UHF* low dose UHF EQD2 < 100 Gy1.5 (α/β = 1.5), *H-UHF* high dose UHF EQD2 ≥ 100 Gy1.5 (α/β = 1.5).

*p-value was calculated between UHF and BT ± EBRT.

For GU toxicity, the 3-year and 5-year cumulative incidence rates of grade ≥ 2 late GU toxicities were 7.5% (13.5%) in the HDR + EBRT group, 9% (13.5% at 5 years) in the LDR group, 4.6% (13.4%) in the LDR + EBRT group, 3% (3%) in the L-UHF group, and 11.1% (17%) in the H-UHF group (*p* = 0.117; Fig. 2d). There were no statistically significant differences among the subgroups in terms of accumulated GU toxicity (Table 5).

### Biochemical control and overall prostate cancer-specific survival

**Figure 3:**
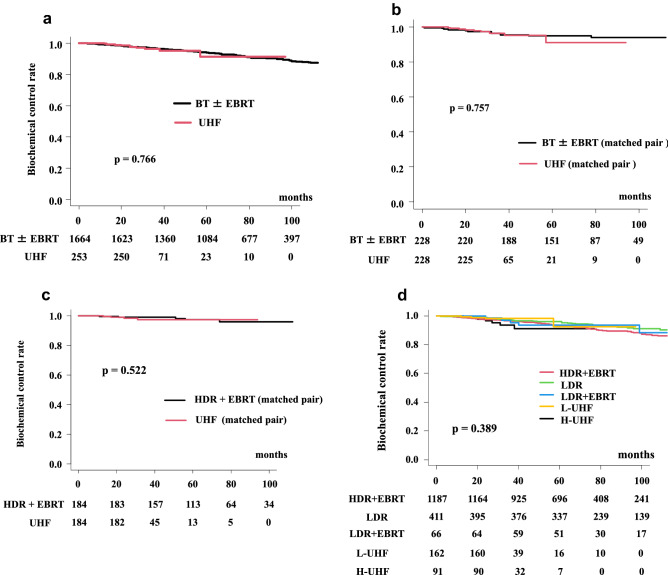
Biochemical control rates between UHF and BT ± EBRT. (**a**) Comparison between UHF and BT ± EBRT in total population. (**b**) Comparison between UHF and BT ± EBRT in matched pair generated by propensity score matching. (**c**) Comparison between UHF and HDR + EBRT in matched pair generated by propensity score matching. (**d**) Comparison among five subgroups.

The number of patients with biochemical failure was 142 in the BT ± EBRT group (8.5%) and 10 in the UHF group (3.95%). The actuarial 3-year and 5-year biochemical control rates were 96.3% (95% CI: 92.7–98.2%) and 96.6% (95% CI: 95.6–97.4%, p = 0.766, Fig. 1) at 3-year, and 91.4% (95% CI: 78.8–96.6%) and 94.0% (95% CI: 92.6–95.1%) at 5-year in the UHF and BT ± EBRT groups, respectively (Fig. 3a).

We generated a well-matched pair (228 and 228 patients; background comparison is shown in Supplemental Table 1) in each group using propensity score matching. The actuarial 3-year and 5-year biochemical control rates were 99.0% (95% CI: 96.0–99.8%) and 99.1% (95% CI: 96.3–99.8%, *p* = 0.164, Fig. 3b) at 3-year, and 92.9% (95% CI: 76.7–98.0%) and 96.1% (95% CI: 92.2–98.0%) at 5-year in the UHF and BT ± EBRT groups, respectively.

The 3-year and 5-year overall survival rates were 99.1% (95% CI: 96.5–99.8%) and 96.6% (95% CI: 90.1–98.8%) for UHF, and 99.4% (95% CI: 98.8–99.7%) and 98.0% (95% CI: 97.1–98.6%) and for the BT ± EBRT groups (*p* = 0.058), respectively. Seventeen and zero prostate cancer-related deaths were observed in the BT and UHF groups, respectively, in this cohort. The 5-year prostate cancer-specific survival rates were 100% and 99.5% (95% CI: 98.9–99.8%, *p* = 0.501) in the UHF and BT ± EBRT groups, respectively.

### Subgroup analysis for Biochemical control and overall prostate cancer-specific survival

For comparison between UHF and LDR only (LDR monotherapy), we included patients with a lower titer intermediate-risk group and low-risk group (Fig. 1). The actuarial 3-year and 5-year biochemical control rates were 97.6% (95% CI: 92.6–99.2%) and 97.2% (95% CI: 95.1–98.5%, *p* = 0.632, supplemental Fig. 1a) at 3-year, and 89.7% (95% CI: 68.6–96.9%) and 95.9% (95% CI: 93.5%-97.5%) at 5-year in the UHF and LDR only groups, respectively.

For comparison between the UHF and LDR + EBRT groups, we included only patients with a high titer of intermediate-risk and high-risk groups. The actuarial 3-year and 5-year biochemical control rates were 94.8% (95% CI: 87.5–97.9%) and 95.2% (95% CI: 85.8–98.4%, *p* = 0.871, supplemental Fig. 1b) at 3-year, and 94.8% (95% CI: 87.5–97.9%) and 93.6% (95% CI: 83.8%-97.5%) at 5-year in the UHF and LDR + EBRT groups, respectively.

For comparison between UHF and HDR+EBRT, the actuarial 3-year and 5-year biochemical control rates were 96.3% (95% CI: 92.7–98.2%) and 96.5% (95% CI: 95.2–97.4%, *p* = 0.962, supplemental Fig. 1c) at 3-year, and 91.4% (95% CI: 78.8–96.6%) and 93.2% (95% CI: 91.4%-94.6%) at 5-year in the UHF and HDR+EBRT groups, respectively.

We generated well-matched pairs in the comparison between UHF and HDR + EBRT (169 patients each; background comparison is shown in Supplemental Table 2) using propensity score matching. The actuarial 3-year and 5-year biochemical control rates were 98.4% (95% CI: 95.0–99.5%) and 98.9% (95% CI: 95.7–99.7%, *p* = 0.522, Fig. 3c) at 3-year, and 97.3% (95% CI: 92.6–99.0%) and 97.3% (95% CI: 92.9%–99.0) at 5-year in the UHF and HDR + EBRT groups, respectively.

For comparison between L-UHF and U-UHF, the actuarial 3-year biochemical control rates were 98.1% (95% CI: 94.3–99.4%) and 93.6% (95% CI: 85.1–97.3%, *p* = 0.139) in the L-UHF and H-UHF groups, respectively (supplemental Fig. 1d). There were no statistically significant differences among the subgroups (Fig. 3d). Among the NCCN risk classifications, the actuarial 3-year biochemical control rates were 100% (L-UHF) and 100% (H-UHF, *p* = 1.0) in the low-risk group; 97.1% (95% CI: 91.2–99.0%), 95.6% (95% CI: 83.3–98.9%, *p* = 0.454) in the intermediate-risk group; and 100%, 88.3% (95% CI: 64.8–96.5%, *p* = 0.109) in the high-risk group. L-UHF showed equivalent outcomes compared with H-UHF.

## Discussion

UHF showed higher GI and equivalent GU toxicity to BT ± EBRT and was largely dependent on the UHF schedule. Additionally, we found an equivalent PSA control rate between UHF and BT ± EBRT, although this was inconclusive due to short follow-up periods. To our knowledge, this is one of the largest cohorts to compare the toxicity of UHF and BT ± EBRT. To reduce bias and amend short follow-up periods, we used the propensity score matched pair analysis, which is the best achievable statistical method and provides a direct comparison of BT ± EBRT and UHF.

Recent advancements in radiotherapy for localized prostate cancer have enabled us to shorten the treatment period using hypofractionations and provide cost effectiveness and patient convenience. In addition to 2.3–3.4 Gy moderate hypofractionation, UHF gained attention for exploiting the low a/b ratio of this tumor and its high radiation fraction size sensitivity^1–6^. The recent HYPO-RT-PC phase 3 trial, which showed non-inferiority of ultrahypofractionation (42.7 Gy/7 fractions for 2.5 weeks) compared with conventional fractionation (78 Gy/39 fractions)^2^. It is anticipated that the efficacy of the UHF treatment schedule will be further validated when the PACE B trial outcome is consolidated and published^24^. Similarly, within our cohort of patients, an excellent biochemical control rate was achieved, which is comparable to HDR ± EBRT, although preliminary.

The HYPO-RT-PC phase 3 trial^2^ reported 28% acute RTOG grade ≥ 2 GU toxicity, and grade ≥ 2 RTOG late GU toxicity was 5% at 5 years, while bowel toxicity was 1% at 5-years. The PACE-B trial reported that the worst acute RTOG toxicity grade ≥ 2 was 23% in GU and 10% in GI^24^. In our UHF data, the worst acute toxicity grade ≥ 2 was 13% in GU and 5% in GI, and accumulated late toxicity grade ≥ 2 was 6% in GU and 5.8% in GI, which concurred with their data. Jackson et al. performed a systemic review and reported that the estimated late grade ≥ 3 GU and GI toxicity rates were 2.0% (95% CI, 1.4–2.8%) and 1.1% (95% CI, 0.6–2.0%) after UHF using SBRT, respectively^4^, which also concurred with our cohort.

In general, BT elevated GU toxicity and reduced GI toxicity compared to EBRT^7^. In addition, although the incidence of acute GU toxicity is tentatively elevated by BT, toxicity was ameliorated by time and cumulative late toxicity did not differ after a few years^7^. For GI toxicity, spacer (SpaceOAR etc.) insertion was found to reduce GI toxicity to almost negligible as no grade ≥ 2 GI events was found in spacer ( +) arms in a randomized controlled trial^25^. This technique could be applied not only in UHF but also in BT ± EBRT. Therefore, we hope that we could reduce GI toxicity in the near future, and the higher incidence of GI toxicity in H-UHF could be reduced with this technique.

As BT can achieve one of the best dose distributions among radiotherapy^7,26^, external beam radiotherapy has made efforts to improve dose distribution using SBRT, intensity-modulated radiotherapy, and image-guided radiotherapy techniques^26^. Several reviews ^27,28^ including three randomized controlled trials^8–10^ have already indicated superiority of BT boost than eternal beam radiotherapy alone. However, BT boost did not show superiority to UHF, and only indicated similarity of BT boost to UHF in low to intermediate risk groups^11–15^. So far, UHF could achieve equivocal outcomes without elevation of toxicity than BT boost in low-to intermediate-risk groups.

In 2018, the American Society for Radiation Oncology (ASTRO), ASCO, and American Urological Association (AUA) evidence-based guidelines stated that extreme hypofractionated 35–36,25 Gy in five fractions (BED 198–211.5 Gy_1.5_) may be offered to patients with low- and intermediate-risk prostate cancer^29^. Royce et al. found that in patients with low to intermediate risk disease treated with UHF, an equivalent dose of 2 Gy per fraction (EQD2) of 71 Gy (31.7 Gy in 5 fractions = BED: 165 Gy_1.5_) achieved a TCP of 90% and an EQD2 of 90 Gy (36.1 Gy in 5 fractions = BED: 209.8 Gy_1.5_) achieved a TCP of 95%^30^. Our data that L-UHF (BED = 198–226 Gy_1.5_) is in line with those of previous reports with a 3-years biochemical control rate of 97.7% (95% CI: 93–99.3%) in the low- to intermediate-risk group.

However, this does not apply for high risk and, most likely, a higher dose is needed^3,31^. Several groups seek better PSA control using higher prescribed doses, especially for intermediate- and high-risk groups^31,32^. In patients with high-risk disease, Royce et al. found that an EQD2 of 97 Gy (37.6 Gy in 5 fractions = 226 Gy1_.5_) can achieve a TCP of 90% and an EQD2 of 102 Gy (38.7 Gy in 5 fractions = 238.4 Gy_1.5_) can achieve a TCP of 95%^3^. Several studies used focal dose escalation with a boost of 38–50 Gy^31,32^. Although our cohort did not show the benefit of H-UHF (BED = 252 Gy_1.5_, with higher GI toxicity without improvement in biochemical control rate [88.3% at 3-years], although with short follow-up periods), further investigation could shed light on the dose escalation for high-risk prostate cancer.

Our study has several limitations. First, the lack of long-term follow-up and the small sample size limits its applicability, with only 25 (9.8%) patients with > 5 years of follow-up in the UHF group, especially in the high-risk group. Longer follow-up may reveal a divergence in toxicity or control rates in the UHF group. Next, the retrospective nature of this study led to an imbalance between the UHF and BT ± EBRT cohorts in terms of baseline characteristics. To mitigate this, we provided a comparative analysis and propensity score-matched analysis. Next, although using a free database is beneficial, its retrospective nature results in an ambiguous recording of the timing of toxicity and tumor control outcomes because of the heterogeneous follow-up periods depending on various institutions and physicians not restricted by protocol. Further studies should be conducted to validate our findings. Finally, for toxicity analysis, other predisposing factors are also important for prediction, including dosimetric factors for organs at risk^33^ and non-dosimetric factors (preexisting symptoms or surgery, transurethral resection of the prostate, anticoagulant use, diabetes mellitus, etc.)^33^.

## Conclusions

UHF showed equivalent efficacy, higher GI and equivalent GU accumulated toxicity to BT ± EBRT, and the toxicity of UHF was largely dependent on the UHF schedule.

## Supplementary Information

Supplementary Information 1.

Supplementary Information 2.

## Data Availability

The data of UHF for this manuscript can be obtained from the public data base on reasonable request [19] and LDR data can be obtained from the author upon reasonable request.
