# Supplementary material for: Comparison of toxicities between ultrahypofractionated radiotherapy versus brachytherapy with or without external beam radiotherapy for clinically localized prostate cancer
Source: Sci Rep. 2022 Mar 23;12:5055. doi: 10.1038/s41598-022-09120-0 (PMC8942991; doi:10.1038/s41598-022-09120-0)
Supplement: Supplementary file 2 — Supplementary Information 2. [file 41598_2022_9120_MOESM2_ESM.pptx]

## Slide 1
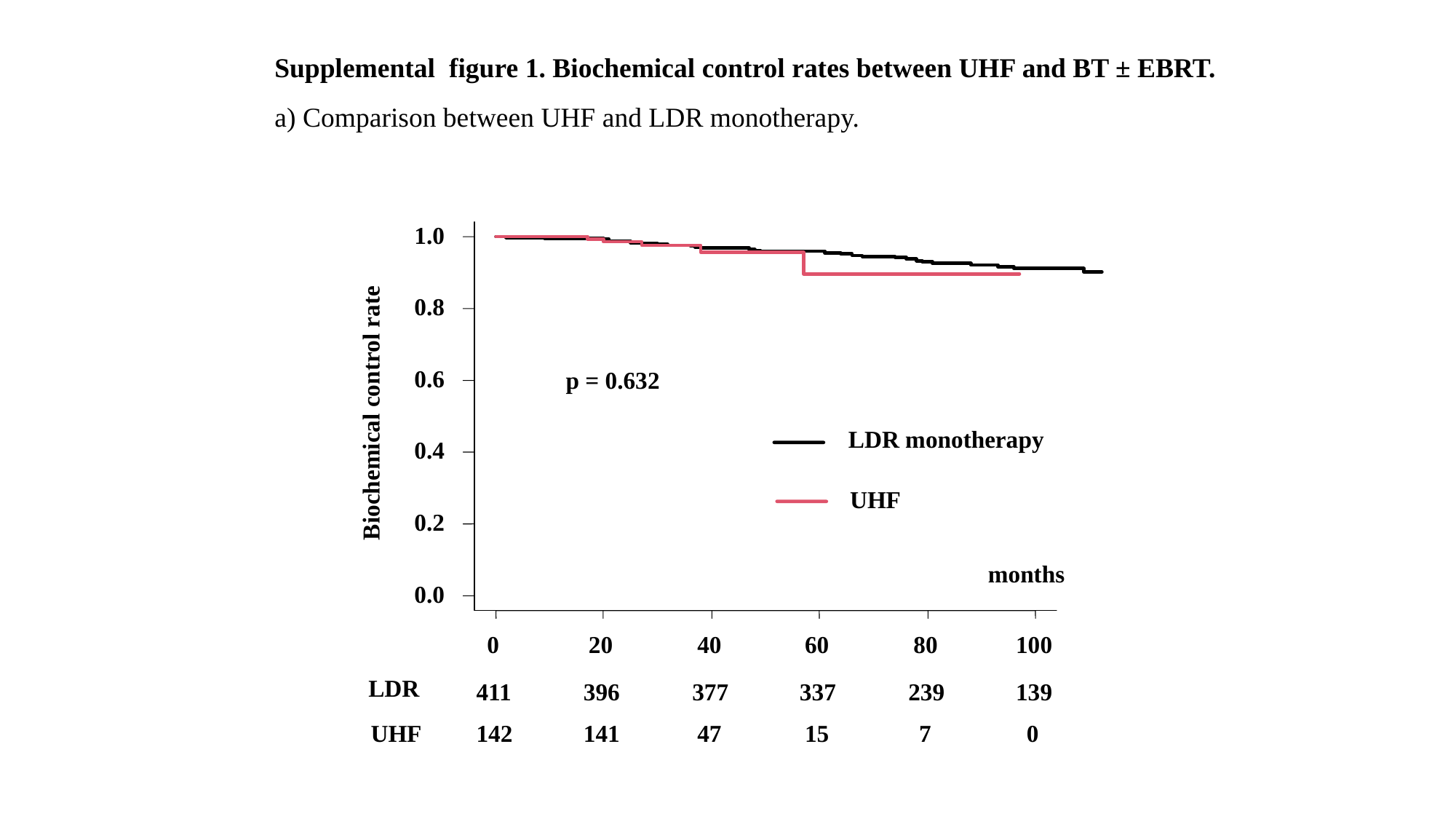

Supplemental figure 1. Biochemical control rates between UHF and BT ± EBRT.
a) Comparison between UHF and LDR monotherapy.
1.0
0.8
p = 0.632
0.6
Biochemical control rate
LDR monotherapy
0.4
UHF
0.2
months
0.0
0
20
40
60
80
100
LDR
411
396
377
337
239
139
UHF
142
141
47
15
7
0

## Slide 2
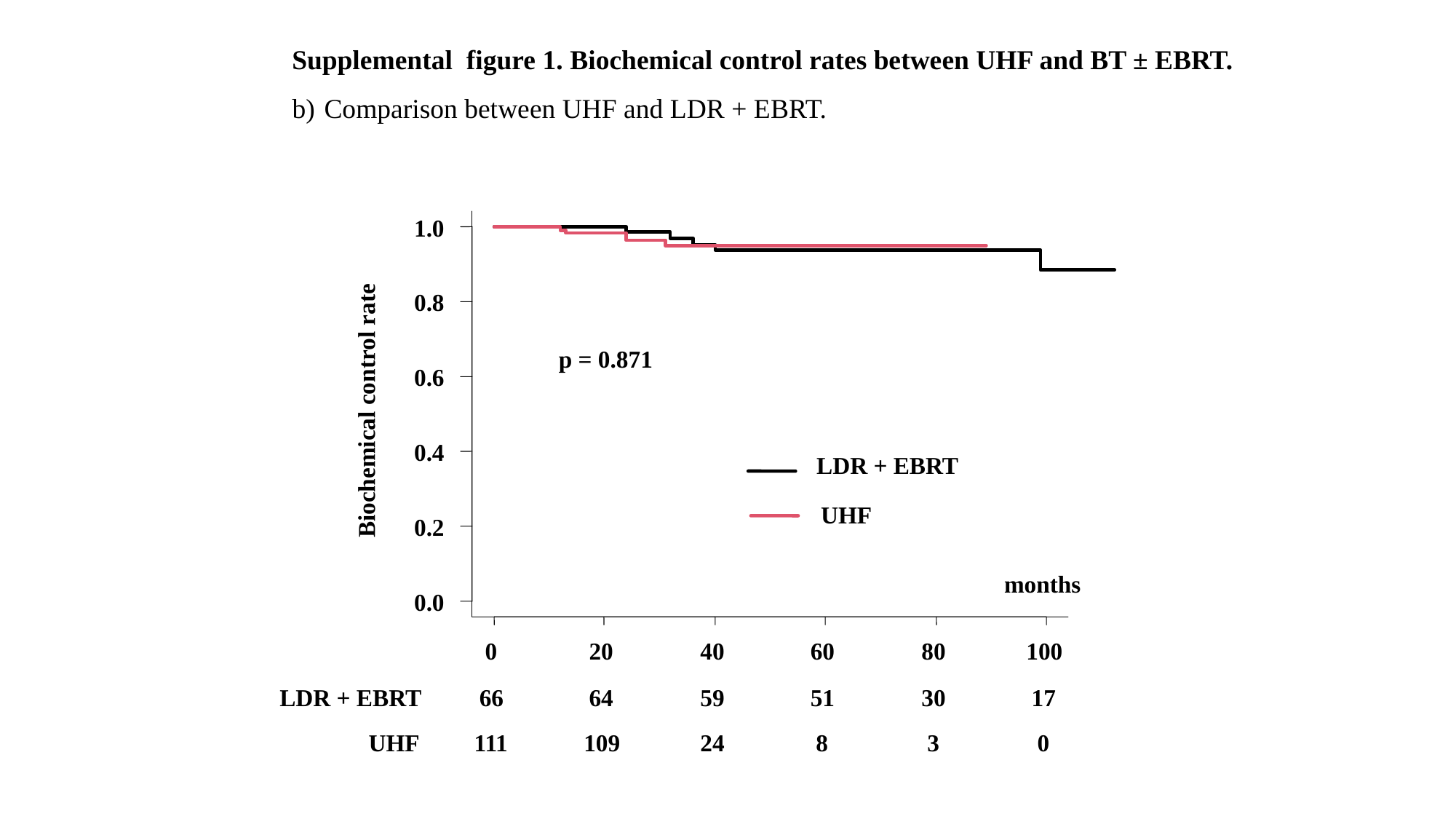

Supplemental figure 1. Biochemical control rates between UHF and BT ± EBRT.
b)	Comparison between UHF and LDR + EBRT.
1.0
0.8
p = 0.871
0.6
Biochemical control rate
0.4
LDR + EBRT
UHF
0.2
months
0.0
0
20
40
60
80
100
LDR + EBRT
66
64
59
51
30
17
UHF
111
109
24
8
3
0

## Slide 3
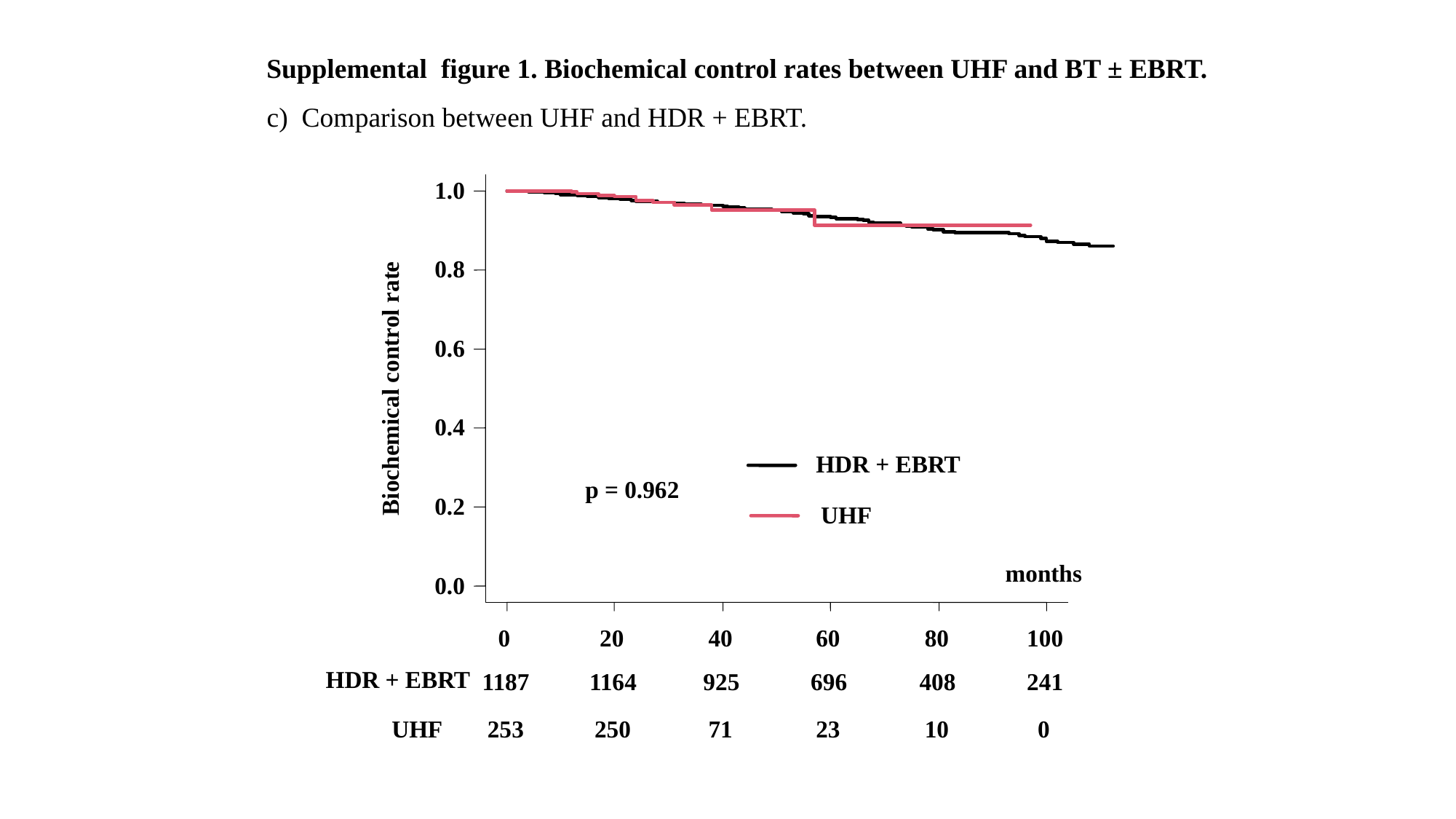

Supplemental figure 1. Biochemical control rates between UHF and BT ± EBRT.
c) Comparison between UHF and HDR + EBRT.
1.0
0.8
0.6
Biochemical control rate
0.4
HDR + EBRT
p = 0.962
0.2
UHF
months
0.0
0
20
40
60
80
100
HDR + EBRT
1187
1164
925
696
408
241
UHF
253
250
71
23
10
0

## Slide 4
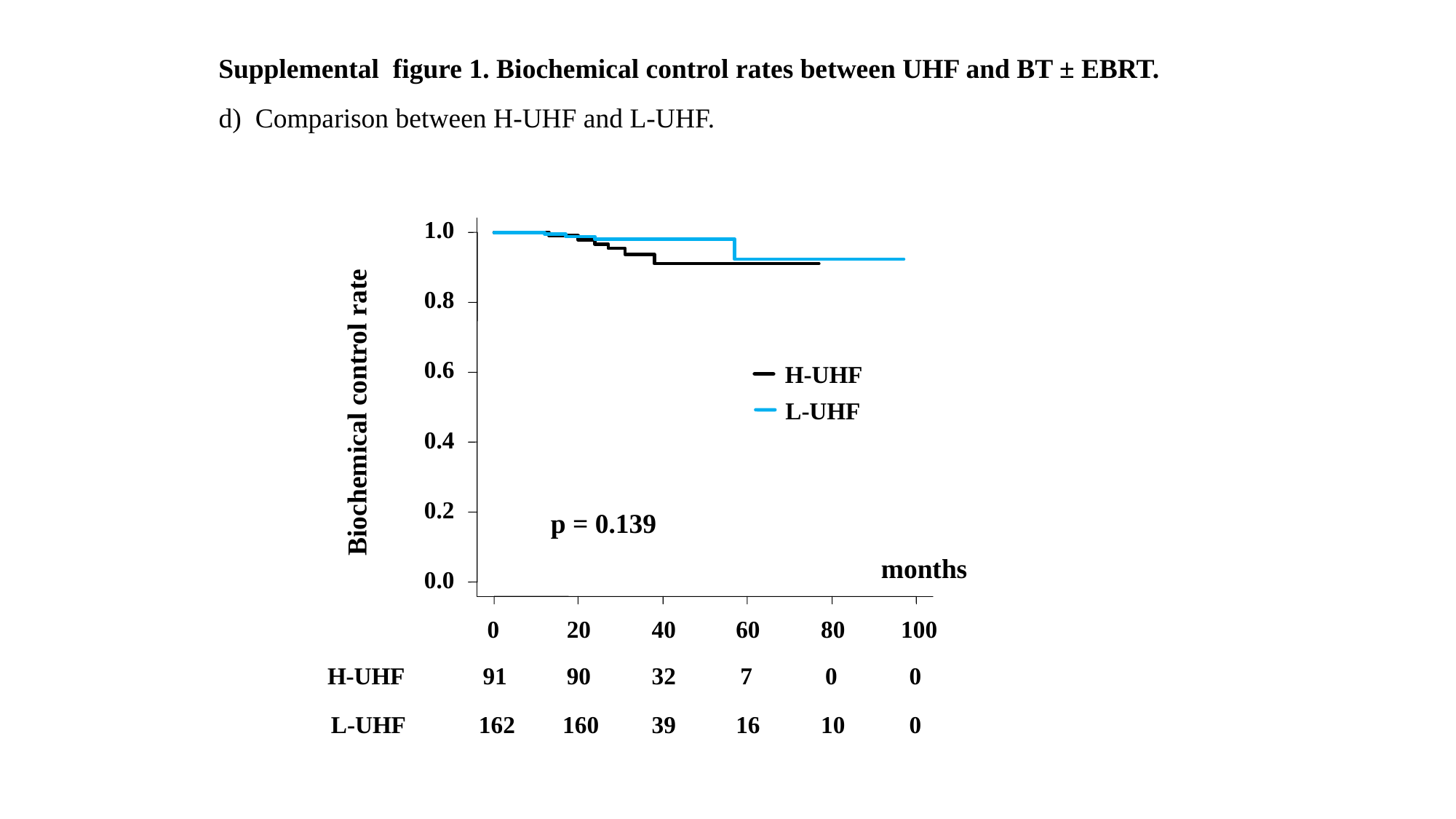

Supplemental figure 1. Biochemical control rates between UHF and BT ± EBRT.
d) Comparison between H-UHF and L-UHF.
1.0
0.8
0.6
0.4
0.2
0.0
0
20
40
60
80
100
H-UHF
91
90
32
7
0
0
L-UHF
162
160
39
16
10
0
H-UHF
L-UHF
Biochemical control rate
p = 0.139
months
